# Self-charging of sprays

**DOI:** 10.1038/s41598-022-21943-5

**Published:** 2022-11-11

**Authors:** Stefan Kooij, Cees van Rijn, Neil Ribe, Daniel Bonn

**Affiliations:** 1 Van der Waals-Zeeman Institute, University of Amsterdam, Science Park 904, Amsterdam, The Netherlands; 2 1Lab FAST, Université Paris-Saclay, CNRS, 91405 Orsay, France

**Keywords:** Surface chemistry, Fluid dynamics, Electrochemistry

## Abstract

The charging of poorly conducting liquids due to flows is a well-known phenomenon, yet the precise charging mechanism is not fully understood. This is especially relevant for sprays, where the spray plume dynamics and maximum distance travelled of a spray dramatically changes for different levels of charging: charging of the droplets makes them repel, thereby preventing drop coalescence and altering the shape of the spray plume. As the charging depends on many factors including the flow and the interactions between the liquid and the nozzle, many models and scaling laws exist in the literature. In this work we focus on perhaps the simplest flow regime, laminar jets created by ultra short channels, and quantify the charging as a function of the different parameters. We present a simple model that collapses all the data for over 4 orders of magnitude difference in streaming currents for various nozzle sizes, flow velocities and surface treatments. We further show that the charging polarity can even be reversed by applying an oppositely charged coating to the nozzle, an important step for any application.

## Introduction

**Figure 1:**
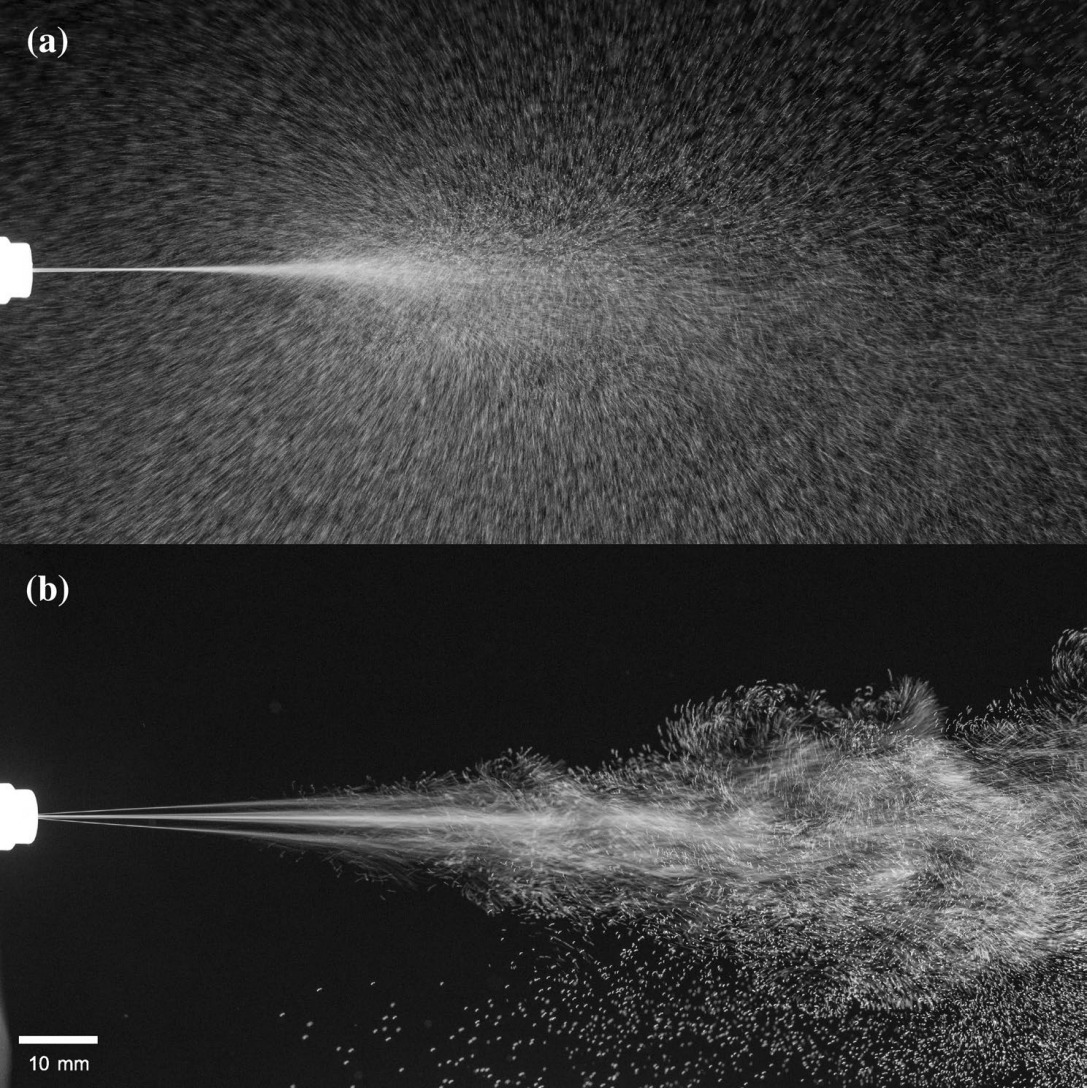
Typical spray plumes with and without self-charging. (**a**) The spray plume generated by a nozzle with 8 holes of radius $R=2 \,\upmu$m using demineralized water. Due to the small spacing between jets, the individual jets cannot be discriminated and appear as one single jet. The self-charging makes the spray plume flare out once the droplets have decelerated significantly. Because of a decrease in coalescence, spray droplets are significantly smaller with self-charging, reducing the maximum travelled distance of the spray plume. (**b**) The same spray now containing small amounts of salt. As there is no charging the spray plume remains narrow and travels a longer distance. Still, due to vortices and complex flow in the surrounding air the spray plume starts to diverge and mix as well.

Spray plume dynamics are important for many applications such as drug delivery^1,2^, health and bodycare, perfumes, agriculture, but also in the spreading of pathogens such as SARS-CoV-2^3,4^. When poorly conducting liquids are used, interaction of the flow with the nozzle can lead to self-charging. If droplets are large, this effect can often be ignored. For fine sprays however, the droplets are small enough such that the electric charges can have a significant impact on the droplet size and the dynamics of the spray cloud (Fig. 1). Such small drops are encountered in many practical situations, in particular drug delivery, inkjet printing or chromatography. It is therefore essential to understand when this effect takes place and to be able to predict the magnitude of the acquired charge.

Charge separation due to liquid flow has been widely studied in various contexts, such as energy production^5,6^, sliding droplets^7^, erosion due to liquid flow^8^, and risks of fire and explosion in the transport of hydrocarbons through metallic pipes^9–12^. The phenomenon of charging of poorly conducting liquids flowing through pipes and orifices is therefore well-known, and as a consequence a number of theories has been developed^12–16^. Still, a clear agreement between theory and experiments is often missing, which can partly be attributed to the complicated dependencies of the charging on the experimental settings. In this work we therefore focus on a clean model situation, i.e. jets and sprays produced by laminar flow through ultra short channels, and develop a theory that agrees extremely well with all of the experiments.

Models for electrokinetic charging are generally based on the fact that when a material comes into contact with a liquid, it will release or absorb some ions, leaving behind a charged surface. With counter-ions moving towards the surface an electrical double layer is formed^17^. The charging that occurs due to the liquid flow is then explained by the overlap between this electrical double layer and the velocity gradient of the flow profile near the wall (Fig. 6). The net transport of charge leads to the so called streaming potential or current. Consequently, the charging strongly depends on the flow profile near the interface, and different behaviours are to be expected for different flow regimes, i.e. laminar versus turbulent and short versus long channels. Indeed, various scaling laws and phenomenological models can be found in the literature, especially for the dependence of the streaming current, $I_{s}$, on the average liquid flow velocity ${\bar{v}}$, which varies from a linear dependence for laminar parabolic flow profiles^18^, to quadratic in turbulent cases^6,19^. Selection of the correct physical model is often complicated by the fact that the charging is strongly affected by the surface chemistry, which can depend on many factors such as pH, cleaning protocols, ageing, and contamination, but can also change due to the liquid flow itself^8^. These complicating factors explain why most research does not allow to discriminate between competing models, that often differ only slightly in scaling exponents.

For most practical nozzles, the channel length is short compared to the hole diameter, as this reduces frictional losses and lowers the minimal acquired pressure to produce a spray. The flow profile in short channels follows a so called top-hat shape, i.e. a constant bulk velocity up to some boundary layer flow near the wall of thickness $\delta x$. In such cases, one can show that the streaming current is inversely proportional to the thickness of this boundary layer, i.e. $I_{s} \sim 1/\delta x$^12^. However, there is no consensus on what gives the thickness of the boundary layer. Duffin et al. performed experiments of microjets of sizes 5–20 $\upmu$m formed with Pt/Ir electron microscopy apertures, and used a boundary layer scaling $\delta x \propto R Re^-7/8$, with *R* the nozzle size and *Re* the Reynolds number as defined below^5,6^. Although this appeared to give a reasonable description of their measured streaming currents, the expression for $\delta x$ is that for the laminar sublayer thickness for turbulent flows through long pipes^20,22^. On the one hand, their inlet length is not large enough to create fully developed turbulence, and on the other hand, the nozzle sizes are so small that it is unlikely that the flow is turbulent at all. Likely for these reasons, Faubel et al. used a different scaling, namely, the Prandtl layer thickness which scales as $\delta x \sim L \sqrt{Re^{-1}}$ with *L* the characteristic channel length, for exactly the same nozzles^21^. They also arrived at a reasonable description of their data, showing that there is no consensus on the thickness of the boundary layer even for the arguably most relevant case of laminar flow through short channels. In this paper, we perform a systematic experimental study over a much larger range of parameters than the previous works, and simultaneously solve the boundary layer thickness problem. We find that the boundary layer in this case scales as $\delta x \sim R \sqrt{Re^{-1}}$, a result that describes our measured streaming currents extremely well.

**Figure 2:**
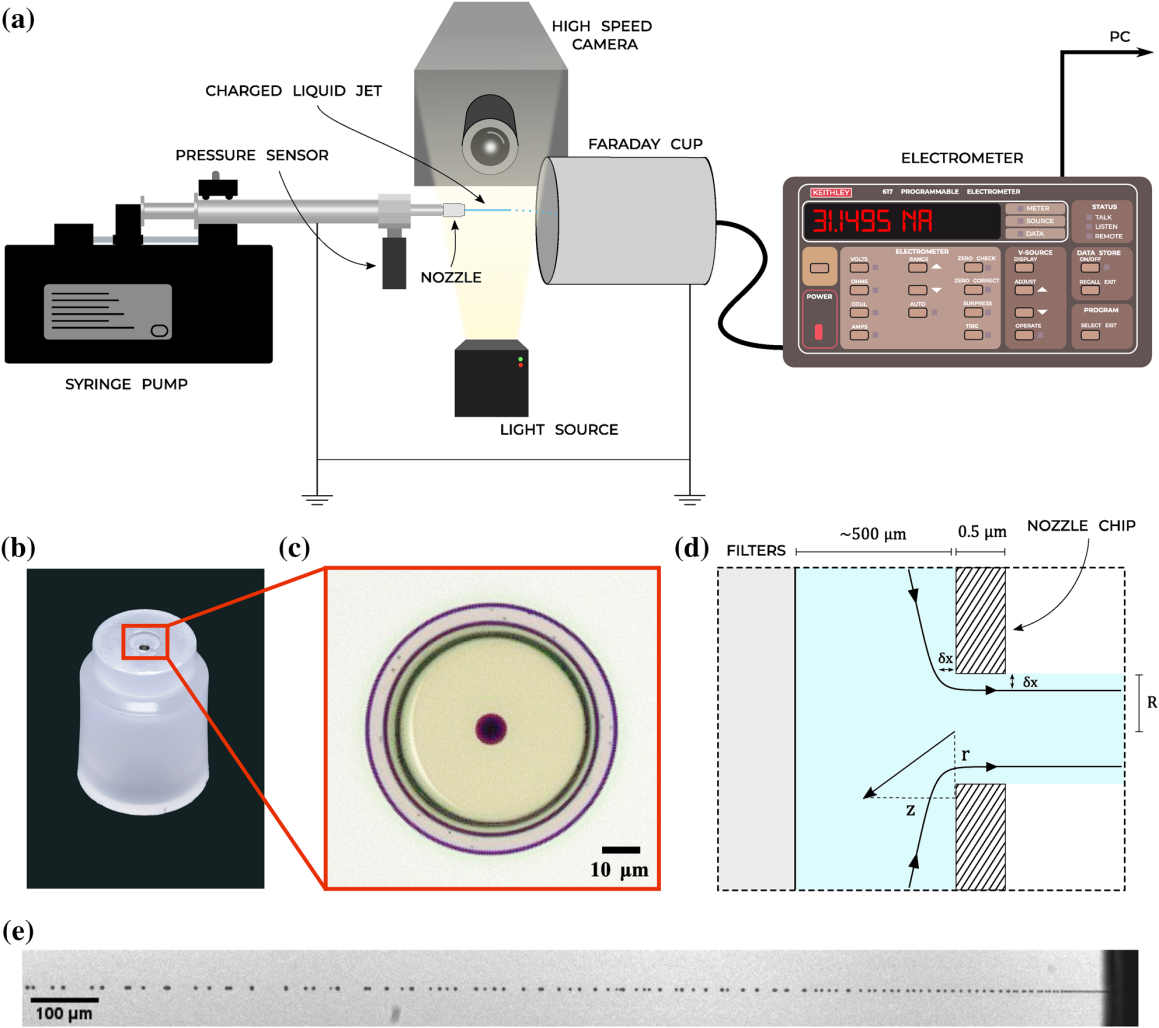
(**a**) Measuring setup. Liquid flow is generated using a Harvard Apparatus ULTRA CP syringe pump and an 8m L stainless steel syringe from Darwin Microfluidics. The grounded syringe is connected to a pressure sensor (U5600-000005-300PA TE Connectivity Measurement Specialties). The streaming current is measured by spraying the charged jet into a Faraday cup. The outside of the Faraday cup is grounded and the inner and outer conducting layers of the cup are connected through a coaxial cable with the Keithley 617 electrometer. Current measurements can be logged simultaneously with the pressure sensor output. To connect the Keithley 617 with the computer a Prologix GPIB-USB connector is used. For the direct velocity measurements of the jets, the jets are filmed using a Phantom TMX 7510, with frame rates upto 870,000 fps. Backlighting is used to illuminate the jets. (**b**) The polypropylene adapter holding the 1 mm $\times$ 1 mm silicon nozzle chip. (**c**) A microscope image of a nozzle chip with a hole radius of 4 $\upmu$m. (**d**) Schematic of the liquid flow through the nozzle. The liquid first passes a filter and continues towards the orifice. The orifice consists of a circular hole in a 0.5 $\upmu$m layer of silicon nitride, with radius *R*, between 2 and 16 $\upmu$m. A boundary layer of thickness $\delta x$ is formed near and in the nozzle hole. (**e**) Liquid jet formed from a $R=8$ $\upmu$m jet, with a liquid velocity of 12 ms$^{-1}$.

In this paper, we explore the electrokinetic charging of sprays consisting of micrometer-sized jets produced by ultra short orifices. This allows us to perfectly quantify the flow parameters. In addition, as the effect of charging is difficult to examine with an array of jets, we mostly focus on single jets. The nozzles used to create the jets consist of a small silicon chip with a circular hole in a 0.5 $\upmu$m thin layer of silicon nitride (Fig. 2b,c). Due to the small size of the orifice, all measurements correspond to the laminar flow regime. The short channel length ensures a plug flow like velocity profile. We measure the streaming current for hole radii between $R = 2$ $\upmu$m and $R = 16$ $\upmu$m and liquid velocities up to 80 ms$^{-1}$. A theoretical prediction is given and is found to match experimental data for over 4 orders of magnitude difference in streaming current.

## Experiments

For the experiments we use spray nozzles consisting of 1 mm $\times$ 1mm silicon chips, with single circular holes of radii $R = 2, 4, 8$ and 16 $\upmu$m (Fig. 2b–d). The silicon chip contains one or multiple cavities of 50 $\upmu$m in diameter up to a 0.5 $\upmu$m thin layer of silicon nitride, the so called membranes. The holes of the nozzle are etched within this thin layer. The length of the channel, *L*, is therefore always the same for each nozzle, and much smaller than the critical length needed to form a parabolic flow profile ($L/R \sim 0.06 Re \sim 40$). The chip is mounted in a polypropylene adapter with a luer fit, and has a small porous filter in front of the nozzle chip. Despite considerable effort to use ultra clean liquids, there are always some contaminants such as single-celled organisms that can easily clog the smallest nozzle holes, therefore requiring the use of prefilters.

The electrical current is measured by spraying the charged jet into a Faraday cup, which is grounded and connected to a Keithley 617 electrometer via a triaxal cable. The Keithley 617 is capable of measuring currents as low as 0.1 fA, however for our experiments no currents were measured below 10 pA. The liquid flow is maintained with a Harvard Apparatus ULTRA CP syringe pump using an 8 mL stainless steel syringe from Darwin Microfluidics. This setup allows to generate pressures up to 40 bar, resulting in jet velocities as high as 80 ms$^{-1}$. The metallic syringe is also connected to ground, so that no charge build-up can occur.

The velocity of the jet was measured using two methods. For the first method we used image analysis to track the trajectories of the micrometer-sized droplets generated by the breakup of the liquid jets. The jets were filmed at the nozzle exit, using a Phantom TMX 7510 camera. Since the droplets are small and the jet velocity high, the required frame rate was 870,000 fps for the fastest jets. By adjusting the flow rate of the syringe pump, the jet velocity was varied. After each flow rate adjustment, a measuring pause of typically a minute was initiated to allow the flow rate, and therefore streaming current, to stabilize. We attribute the delayed onset of the correct flow rate by the presence of air bubbles in the system, which act as small pressure vessels. Indeed by carefully refilling the syringe avoiding air bubbles, the waiting time can be reduced. After stabilization, each image recording was initiated while simultaneously reading off the current on the electrometer.

For the second method we use a pressure sensor (U5600-000005-300PA TE Connectivity Measurement Specialties), to read out the pressure along with the electrical current, which is logged using the Prologix GPIB-USB, connecting the Keithley electrometer with the computer. As the nozzle consists of a small porous filter followed by an orifice, the velocity can be directly calculated from the pressure using Bernoulli’s principle, plus a correction term for the flow through the filter (Darcy’s law). We find that this indirect measurement of the jet velocity perfectly coincides with the measurements from the pressure sensor, as well as the calculated velocity from the syringe pump flow rate and the nozzle hole diameter.

As the direct velocity measurements using high speed photography cannot be performed continuously, it is difficult to measure the streaming current when the flow rate approaches zero. However, when the syringe pump is discontinued, the liquid pressure does not go to zero directly, which is likely due to air bubbles as mentioned previously. During this final slow pressure drop, both the fluid pressure and electrical current can be recorded up to the moment the pressure is insufficient to support the formation of a jet and the measurement is discontinued. This method allows to measure the streaming current for very low flow rates.

## Results

**Figure 3:**
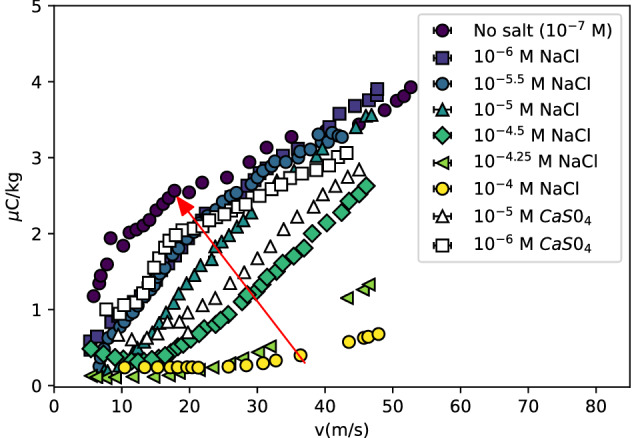
Charging efficiency for different salt concentrations in water. Salt changes the conductivity of the liquid as well as the electrical double layer. The arrow indicates decreasing salt concentrations (NaCl), showing that for salt concentrations higher than $10^{-4.25}$ M, charging becomes insignificant. The white markers show the multivalent salt CaSO_4_, which should have a larger screening effect. Though the charging is less efficient than for NaCl, the effect is not very strong.

The electrokinetic charging only becomes apparent when poorly conducting liquids are used. Due to the self-ionization of water, pure water always contains some ions, but at a very low concentration ($10^{-7}$ M), giving pure water a conductivity of only 5 $\upmu$S cm$^{-1}$. Figure 3 shows the charging efficiency measured for a $R=8$ $\upmu$m nozzle, for different concentrations of NaCl. The results demonstrate that for water with salt concentrations higher than $10^{-4.25}$ M, the electrokinetic charging starts to disappear. The ions present in the fluid not only change the conductivity of the liquid, but also the screening length of the electrical double layer, i.e. the shielding of the electric surface charge. The colorless markers in Fig. 3 show the behaviour for a multivalent salt CaSO_4_, which should have a stronger screening effect. Though the curves for the multivalent salt are a bit below the equally concentrated NaCl solutions, the effect is small.

**Figure 4:**
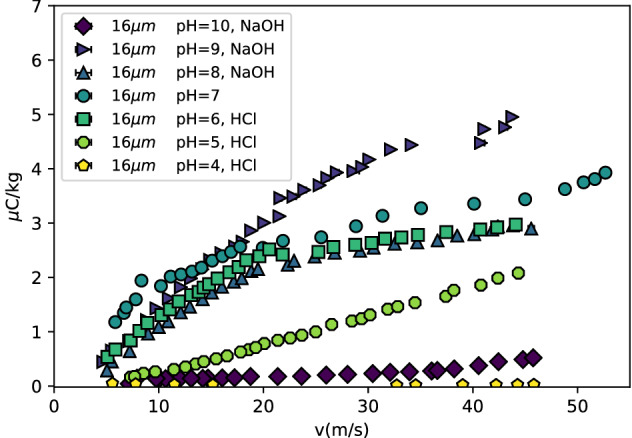
Charging efficiency for different pH levels in water. For very high or low pH the charging efficiency decreases, as can be expected from the increase in conductivity. For pH=9 however, the charging efficiency seems to be the highest. As silicon dioxide is a very weak acid (orthosilicic acid ), the zeta potential increases with higher pH^23^, which easily explains the optimal pH is higher than neutral.

The charging is strongly affected by the surface chemistry, or zeta potential, of the orifice. The orifice surface consisting of silicon dioxide, is a weak acid (orthosilicic acid), and therefore has a decreasing, i.e. more negative, zeta potential for higher pH values^23^. A more negative zeta potential would therefore increase the streaming current. However, the conductivity also increases for higher pH. It is therefore expected that the optimal pH for silicon dioxide is somewhat higher than neutral. Figure 4 shows the charging efficiency for a $R=8$ $\upmu$m sized jet for water of different pH levels and liquid velocities. When the pH is very high or low, the charging diminishes. As expected, the optimal pH is higher than neutral and seems to be close to pH = 9.

**Figure 5:**
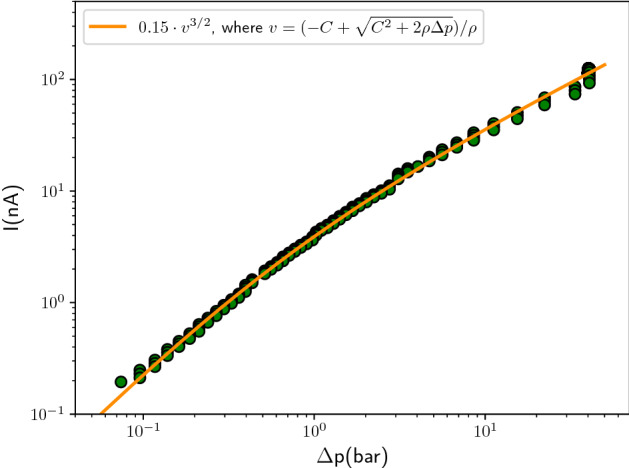
Example of the streaming current $I_{s}$ against the pressure drop $\Delta p$. Following Eq. (12) the streaming current scales as $I_{s}\sim v^{3/2}$, solving Eq. (1) for $v$ allows one to express $I_{s}$ in terms of $\Delta p$. The orange line shows that this gives an excellent prediction of the experimental results, for a certain value of *C*, which is determined by the filter’s porosity and the liquid viscosity. This causes *C* to differ slightly between experiments.

As the nozzle hole diameter is small and the length of the channel considerably smaller than the hole size, the flow through the hole is a plug flow up to a small boundary layer, such that liquid flow through the nozzle chip only depends on the liquid density and pressure drop according to Bernoulli’s principle. This means that viscous losses are very small as required by the small Reynolds numbers (see following section). However, to avoid clogging due to impurities, a porous filter is placed in front of the nozzle chip. The flow through the filter is described by Darcy’s law, which states that the flow rate *Q* scales linearly with the pressure drop over the filter. The total pressure drop can therefore be calculated through Bernoulli’s principle plus a small linear correction term to account for the presence of a filter, such that

1 $$\begin{aligned} \Delta p = \frac{1}{2}\rho v^{2} + C v, \quad \quad C = \frac{A_{2}\mu L}{A_{1}k} \end{aligned}$$

where $\mu$ is the liquid viscosity, *L* the length of the filter, *k* the nozzle’s filter porosity and $A_{2}/A_{1}$ the ratio between the area of the nozzle hole and the area of the filter. According to Eq. (12) the streaming current should scale as $I_{s} \sim v^{3/2}$. Solving for $v$ in Eq. (1), the streaming current can be expressed in terms of $\Delta p$ as

2 $$\begin{aligned} I_{s} \sim v^{3/2} \sim \left[ \frac{-C+\sqrt{C^{2}+2\rho \Delta p}}{\rho }\right] ^{3/2}, \end{aligned}$$

where *C* is a parameter set by the liquid viscosity and by the properties of the filter such as its porosity (see Eq. 1). Figure 5 shows the streaming current, $I_{s}$, against the pressure drop, $\Delta p$, for a typical experiment, with the orange line indicating the prediction following Eq. (2). The line shows an excellent agreement for a given value of *C*, which varies between experiments, due to differences in liquid viscosity and porosity of the filter. Especially the latter can change drastically through clogging of the filter, though for fresh nozzles the differences are of the order of 50%. Equation (2) therefore allows one to convert pressure measurement data into liquid velocities.

## Theory

### Streaming current

**Figure 6:**
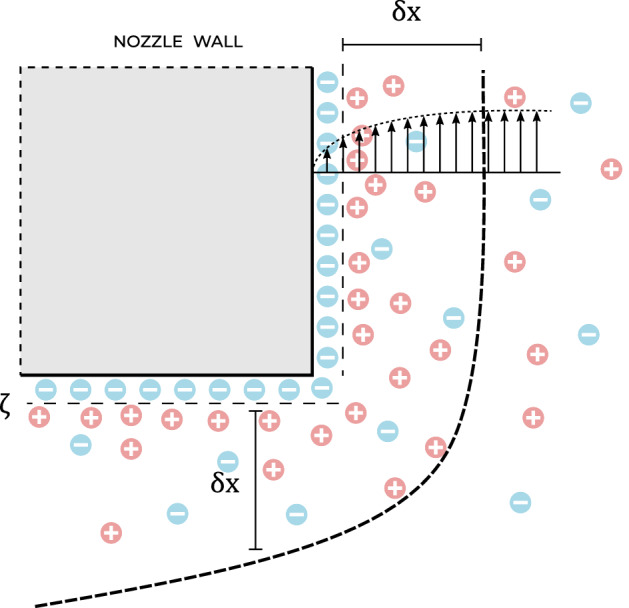
Hydrodynamic boundary layer velocity profile interacting with the electrical double layer. When a material comes into contact with a liquid it will release or absorb some ions according to its zeta potential. For charge neutrality counterions will move towards the surface to form a diffusive double layer. For laminar flow through short channels, liquid flow goes from zero near the wall up to the bulk velocity $v$ over some boundary layer thickness $\delta x$. The charging depends on this layer thickness, which scales as $\delta x \sim R\sqrt{Re^{-1}}$.

On the liquid-wall interface, there is always some exchange of ions, the amount of which sets the created potential difference, or zeta potential (Fig. 6). Due to charge neutrality, counterions will move towards the surface to form a diffusive double layer. For example, as in case of the nozzle chip that contains a layer of silicon dioxide, the silanol hydroxyl groups will act as a weak acid when in contact with water and release some H+ ions at the interface. The charging is then due to the interaction of the liquid flow and this electrical double layer, transporting a net charge. The streaming current $I_{s}$, can then be calculated by integrating the liquid velocity profile $v$(*r*) and the charge density distribution $\rho (r)$ as

3 $$\begin{aligned} I_{s} = \int _{0}^{R}v(r)\rho (r)2\pi r dr. \end{aligned}$$

For short channels the velocity profile $v$(*r*) is a plug flow, up to some boundary layer flow of thickness $\delta x$, where the liquid velocity goes from the bulk velocity $v$ to zero at the interface (Fig. 6). Davies^12^ showed that by taking as a velocity gradient, $v/\delta x$, the streaming current can be approximated by

4 $$\begin{aligned} I_{s} \approx -\frac{R v \zeta \epsilon }{2\delta x}, \end{aligned}$$

where $\zeta$ is the zeta potential, $\epsilon$ the electric permittivity of the liquid, $v$ the liquid velocity, *R* the radius of the nozzle hole and $\delta x$ the boundary layer thickness.

Though liquid velocities are up to 80 ms$^{-1}$, due to the small nozzle hole radii Reynolds numbers

5 $$\begin{aligned} Re = \frac{\rho v R }{\mu }, \end{aligned}$$

are not higher than 400 in our experiments. The liquid flow can thus be expected to be laminar.

The thickness of the boundary layer (Figs. 2d, 6), $\delta x$, near the nozzle hole can be determined using scaling analysis. Let *R* be the spherical radial coordinate measured from the center of the orifice, and let *r* and *z* be cylindrical coordinates such that the start of the hole is $z = 0$. The first step is to write down an expression for the inviscid flow external to the boundary layer. This flow is approximately a purely radial inflow towards the orifice, which can be treated as a point sink with volumetric strength *Q*. The associated radial velocity is

6 $$\begin{aligned} U_{R}= -\frac{Q}{2\pi R^{2}}, \end{aligned}$$

which is then also the velocity at the boundary layer. The flow in the boundary layer satisfies the steady boundary layer equation

7 $$\begin{aligned} u_{z}\partial _{z}u_{r} + u_{r}\partial _{r} u_{r} = v \partial _{zz}^{2}u_{r}, \end{aligned}$$

where $u_{z}$ and $u_{r}$ are the cylindrical components of the velocity inside the boundary layer. There is no pressure gradient term in Eq. (7), because the pressure associated with the external flow is identically zero. Now according to standard boundary-layer theory, the two terms on the left-hand side of Eq. (7) are of the same order of magnitude. Therefore we have

8 $$\begin{aligned} u_{r}\partial _{r} u_{r} \sim v \partial _{zz}^{2}u_{r}. \end{aligned}$$

Scaling $\partial _{r}\sim -r^{-1}$ and $\partial _{z} \sim \delta x^{-1}$, we obtain

9 $$\begin{aligned} -\frac{u_{r}^{2}}{r} \sim v \frac{u_{r}}{\delta x^{2}}. \end{aligned}$$

Solving for $\delta x$ and setting $u_{r}\sim U_{r}$, we obtain

10 $$\begin{aligned} \delta x \sim \left( \frac{2\pi v r^{3}}{Q}\right) ^{1/2}. \end{aligned}$$

The boundary layer therefore becomes progressively thinner as one approaches the hole, due to the strong acceleration of the external stream as *r* decreases. Now the boundary layer thickness at the edge $r = R$ of the hole is

11 $$\begin{aligned} \delta x \sim \left( \frac{2 v R}{v}\right) ^{1/2} \sim R \;\sqrt{Re^{-1}}. \end{aligned}$$

Using this expression in Eq. (4) gives for the streaming current

12 $$\begin{aligned} I_{s} = -\frac{v \zeta \epsilon }{2 \sqrt{Re^{-1}}}. \end{aligned}$$

This expression for the streaming current differs slightly from what is often used in the literature^5,6,12^, as most authors use for $\delta x$ the result of Rouse and Howe^20^ for the laminar sublayer thickness in a turbulent flow, which equals

13 $$\begin{aligned} \delta x \approx 116 \;R\; Re^{-7/8}, \end{aligned}$$

though Faubel^21^ uses the Prandtl layer thickness which scales as $\delta x \sim L \sqrt{Re^{-1}}$, where *L* is the characteristic channel length. However, none of the experimental results in literature seems to be able to fully discriminate between the different scaling laws nor do the expressions used for the layer thickness seem to be applicable for such short channels in a laminar flow regime. In this work however, we find that the data can only be described by taking the derived boundary layer thickness $\delta x \sim R \;\sqrt{Re^{-1}}$, which gives a correct prediction for over 4 orders of magnitude difference in streaming current.

### Spray plume dynamics and droplet charge

To understand the effect of the electric charges on the spray droplets, we can start by estimating when the charging efficiency becomes sufficient to prevent droplet coalescence. From high speed images we observe that droplets generated from jets have slightly different velocities, which as they travel in the same direction, is the main driving force for coalescence. We find that the relative velocity between droplets is normally distributed around a mean of approximately $\Delta v \approx v/100$. It should be noted though, that the velocity distribution depends on the nature of the perturbations that cause the instability of the jet, which might be very different, e.g. when the perturbation are not random, but due to an external source. Droplet coalescence will not occur if the potential energy due to the electric charge is higher than the kinetic energy of the approaching droplets. If we take as the interdroplet distance the Rayleigh wavelength, the potential energy between two equally charged droplets is

**Figure 7:**
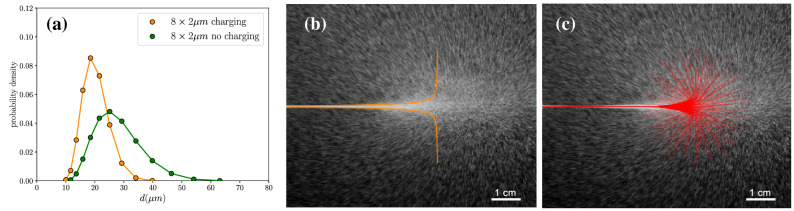
Measured droplet size of a 8$\times R=2$ $\upmu$m spray nozzle, i.e. 8 holes of radius 2 µm, and numerical models for the expansion of the spray cloud due to self-charging. (**a**) droplet size distributions measured with laser diffraction (Malvern Spraytec) for a 8 $\times 2$ $\upmu$m spray nozzle at a distance of approximately 0.05 m from the nozzle, with and without self-charging (see also Fig. 1). Sodium chloride is added to produce uncharged droplets. Droplet sizes are smaller in the case of self-charging, though droplet coalescence is not completely prevented. (**b**) the predicted divergence of the spray cloud according to Eq. (22). (**c**) numerical simulation of 200 droplet trajectories projected on the x-y plane.

14 $$\begin{aligned} U_{e} = k_{e} \frac{q^{2}}{9 R}, \end{aligned}$$

where $k_{e}$ is the electrostatic constant, *R* the radius of the nozzle hole and *q* the charge of each droplet. The charge density of a jet with velocity $v$ is

15 $$\begin{aligned} \rho _{q} = \frac{I_{s}}{\pi R^{2} v}. \end{aligned}$$

For Rayleigh breakup the droplet radius is $R_{d} \approx 1.89 R$, which gives for the droplet charge

16 $$\begin{aligned} q = \rho _{q} \frac{4}{3}\pi R_{d}^{3} = \frac{4 I_{s}}{3 v}\frac{R_{d}^{3}}{R^{2}} \approx \frac{I_{s}}{v}\cdot 9 R. \end{aligned}$$

The potential energy can then be expressed in terms of the streaming current as

17 $$\begin{aligned} U_{e} \approx k_{e} \frac{I_{s}^{2} 9 R}{v^{2}}. \end{aligned}$$

To prevent droplet coalescence for the fastest droplets, the electrical potential should be much larger than the average kinetic energy of the approaching droplets, i.e. $U_{e}/E_{kin}\gg 1$. The kinetic energy is given by

18 $$\begin{aligned} E_{kin} = \frac{1}{2}\frac{4}{3}\pi R_{d}^{3}\rho \left( \frac{v}{100}\right) ^{2} \approx 1.4\cdot 10^{-3} \rho R^{3} v^{2}, \end{aligned}$$

where we use that the droplet radius $R_{d}$ is approximately 1.89*R* for Rayleigh breakup. The criteria for droplet coalescence suppression for water then becomes

19 $$\begin{aligned} \frac{U_{e}}{E_{kin}} = 6.4\cdot 10^{3}\; k_{e} \frac{\zeta ^{2}\epsilon ^{2}}{\mu v R} \approx \frac{3\cdot 10^{-4}}{v R} \gg 1, \end{aligned}$$

where we use $\zeta \approx 100$ mV (see following section). This shows that the larger the jet radius, the smaller the effect of the charging is on the droplet dynamics, as can be expected. For a spray of hole radius $R=2$ $\upmu$m and a typical spray velocity of $v =20$ ms$^{-1}$, $U_{e}/E_{kin} \approx 8$, so that one can expect droplet repulsion to become relevant. Indeed, droplet sizes measured at equal distances using a laser diffraction technique (Malvern Spraytec), are significantly smaller when there is self-charging (Fig. 7a). Still, as compared with the nozzle radius, *R*, an average of 15 droplets must have merged to reach the measured median droplet size. A more detailed measurement of the droplet velocity distribution would allow to evaluate the coalescence criteria for the whole spray. Such detailed measurements are however beyond the scope of this paper.

As is clear from Fig. 1, the self-charging has a significant impact on the spreading of the spray plume. To estimate the expansion of the spray plume, ignoring other aerodynamic effects such as vortices, we explore a simple model for the contours of the spray plume and perform a direct numerical simulation of an array of charged droplets. Let $\vec {r}(z)$ be the radial position from the center of the jets and *z* the axial distance from the start of the nozzle. The electric field due to a stream of charged droplets can be estimated by viewing the jets as a charged cylinder with a charge density $\lambda$ such that

20 $$\begin{aligned} \vec {E} = \frac{\lambda }{2\pi \epsilon _{0}r(z)}{\hat{r}} = \frac{I_{s}}{2\pi \epsilon _{0}r(z) v_{a}(z)}{\hat{r}}, \end{aligned}$$

where we use $\lambda = I_{s}/v_{a}(z)$, with $v_{a}(z)$ the velocity in the axial direction. The droplet charge can be calculated by using the charge density and droplet volume as in Eq. (15), such that the Coulomb force on a particle is

21 $$\begin{aligned} \vec {F_{c}} = q \vec {E} = \frac{2 I_{s}^{2}}{3 \pi \epsilon _{0}r(z) v_{a}(z)v_{0}}\frac{R_{d}^{3}}{R_{nozzle}^{2}} {\hat{r}}, \end{aligned}$$

where $v_{0}$ is the velocity of the fluid at the nozzle exit. The electric repulsion is opposed by frictional forces, $\vec {F_{s}}$, according to Stokes’ law such that the full equation of motion becomes

22 $$\begin{aligned} m \frac{d\vec {v}}{dt} = \vec {F_{c}}-\vec {F_{s}} = \frac{C_{1}}{r(z)v_{a}(z)}{\hat{r}}-C_{2}\vec {v}, \end{aligned}$$

where $$C_{1} = 2 I_{s}^{2} R_{d}^{3}/(3\pi \epsilon _{0}v_{0} R_{nozzle}^{2})$$ and $$C_{2}=6 \pi \mu _{air} R_{d}$$.

The movement of the droplets in the spray strongly depends on their size, $R_{d}$, as the deceleration or stopping distance of the spray scales as $\sim R_{d}^{2}$. Using the droplet size as expected from Rayleigh breakup would lead to unrealistically short spray plumes. Due to coalescence the median droplet size is significantly larger, resulting in much larger stopping distances of the spray droplets. For the spray nozzle in Fig. 1, the measured median droplet radius is 9.2 $\upmu$m at the point the spray plume starts to diverge (Fig. 7a), i.e. when the radial velocity becomes comparable to the axial velocity, while from Rayleigh breakup one would expect droplets of radius $\sim \;$3.8 $\upmu$m. Using the measured droplet radius for $R_{d}$, the streaming current $I_{s}$, and placing the droplet at the edge of the spray, i.e. $r(z=0)=250$ $\upmu$m, the equation of motion can be solved numerically. Figure 7b shows the predicted contour of the spray plume for this nozzle configuration compared with a picture of the actual spray plume, which demonstrates an excellent agreement.

We also performed a basic numerical simulation of a single jet using the Euler method for integration. As initial conditions, we use the measured droplet size and corresponding interdroplet spacing, i.e. assuming an average of 15 droplet coalescence events to reach the measured droplet size. We simulate a jet by periodically producing droplets, with a maximum of 200 droplets for computational efficiency. To the starting position of each droplet a small noise parameter is added, something that can also be expected in a real experimental set-up. Perfectly aligned droplets would lead to unrealistic artefacts in the electrostatic interaction. The force on each droplet is then the sum of all electrostatic forces and the frictional drag according to Stokes’ law. Figure 7c shows an overlay of the spray plume with the projection of all the 3D trajectories on the xy-plane according to the simulation. Though the real spray consists of multiple jets, this simple model with only a single jet captures the deceleration and the radial spreading of the spray droplets.

**Figure 8:**
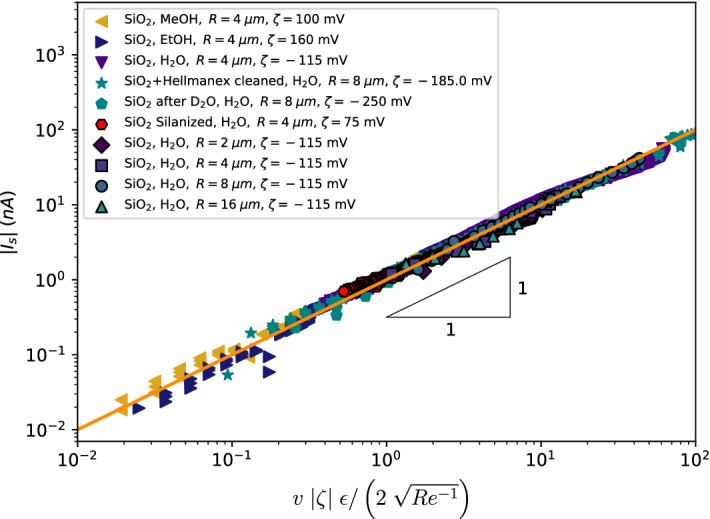
Streaming current against the prediction (Eq. 12). The absolute value of the streaming current, $|I_{s}|$, plotted against the prediction, for different flow rates, nozzle sizes, liquids and surface treatments. A negative zeta potential in this case indicates a positively charged jet and vice versa. When anhydrous ethanol (EtOH) or methanol (MeOH) is used, the polarity is opposite that of water. Mixed with water, the zeta potential is however again negative (not shown in the figure). Cleaning the nozzle prior to the measurement with Hellmanex III leads to a more negative zeta potential. When heavy water is sprayed before measuring the streaming current with normal water, the zeta potential temporarily more than doubles from $\zeta =-115$ mV to $\zeta =-250$ mV. This indicates some kind of incorporation of deuterium atoms on the surface, or the absorption of deuterium oxide molecules. By applying a silane coating on the silicon dioxide surface with positively charged end-groups, the zeta potential can be reversed ($\zeta = 75$ mV), leading to a negatively charged jet for water.

## Discussion

We are now in a position to compare theory and experiment. Figure 8 shows the data collapse using Eq. (12) for all measurements, where the liquid velocities were determined by using the pressure sensor data, as well as direct measurements using the high speed camera. We use the absolute value of the streaming current and the zeta potential, so that both polarities fall on the same curve. A positive value for the zeta potential therefore indicates a negatively charged jet (legend Fig. 8). We varied the liquid velocity, nozzle hole diameter, fluid type and the treatment of the nozzle wall surface. The only unknown fitting parameter is the value of the zeta potential, $\zeta$, which depends on the chemical interaction of the liquid with the nozzle surface. Interestingly, we find that many things can have a significant impact on the value of the zeta potential. For example, cleaning the nozzles beforehand with Hellmanex III, makes that the zeta potential decreases from $\zeta = -115$ mV to $\zeta = -185$ mV. Also the use of other liquids can have a temporary effect on the surface chemistry. Curiously, by first flowing heavy water through the nozzle, the zeta potential more than doubles when normal water is subsequently used. This suggests some kind of exchange of deuterium on the surface or the absorption of deuterium oxide molecules. Coupling of the ionized jet with a mass spectrometer together with the use of normal and deuterated molecules, could provide a way to track the exchange of protons so as to give more insight into the chemical interactions at the interface, however this is beyond the scope of this paper. Given the hysteresis effects on the determination of the zeta potential, measurements were carried out after prolonged spraying to reach a steady state.

Besides the effect of density, viscosity and electric permittivity on the change of liquid, the zeta potential depends on the chemical interaction of the liquid with the surface, which especially in the case of SiO_2_ surfaces is notoriously difficult to understand. In water, the silanol groups at the surface of SiO_2_ act as a weak acid, releasing protons at the interface to form a negatively charged surface. The resulting jets therefore have a positive charge (Fig. 8). Alcohols such as methanol and ethanol can, by the presence of a hydroxyl group, both absorb or donate a proton. We find that water/ethanol mixtures lead to positive charging, but when anhydrous methanol or ethanol is used, the polarity changes (Fig. 8). As there must be a cross-over between both polarities, when the ethanol or methanol samples contain only small amounts of water ($\lesssim 10$%), the charging efficiency becomes very low. Predicting the zeta potential in such cases would require the calculation of the acid dissociation constant between the silanol groups of the nozzle wall and the hydroxyl groups of the liquid, for which at the present time there is no available literature.

To further demonstrate that the charging polarity is due to the surface charge, we apply a silane coating with positively charged end-groups. After silanization the polarity with water is reversed, with a zeta potential of $\zeta =$ 75 mV (Fig. 8).

Equation (12) shows that the streaming current only weakly depends on the radius of the hole, i.e. $I_{s}\sim R^{1/2}$. We change the radius of the hole by a factor of 8, and find that the predicted streaming current still holds over a large range of liquid velocities (Fig. 8). As the flow rate scales with $\sim R^{2}$ for a given liquid velocity, the charging efficiency scales as $\sim R^{-3/2}$. Therefore, better charging efficiencies can be achieved by using smaller orifices.

The results so far only dealt with single hole nozzles, while most practical spray applications require a larger flow rate and therefore a nozzle consisting of an array of holes, such as the nozzle in Fig. 1. Though one can expect the streaming current to simply scale with the number of holes, saturation effects could play a role. For a $8\times 4$ $\upmu$m spray nozzle at a flow rate of 300 $\upmu$L  min$^{-1}$ using pure water, we measure a streaming current of $210/8=25.5$ nA per hole, while according to Eq. (12) one expects this to be 20 nA, a difference that can easily be attributed to a small difference in the zeta potential.

## Conclusions

When poorly conducting liquids are sprayed, they can undergo self-charging, an effect that can have a significant impact on the spray plume dynamics and droplet size. This electrokinetic charging is a result of the interaction of the liquid velocity flow profile near the nozzle wall and the electrical double layer, which in turn depends on often complicated surface chemistry. In this work, we explored the charging for a model situation, laminar flow through micrometer-sized orifices, that is amendable to a full theoretical analysis. Contrary to flow through long pipes, the ultra short orifices lead to a plug-like flow profile with a thin boundary layer at the nozzle wall. As the streaming current is due to the overlap between the liquid flow profile and the electrical double layer, one can show that the streaming current is inversely proportional to the boundary layer thickness $\delta x$. We showed that for this case, boundary layer theory predicts that the liquid velocity profile is within a layer thickness of $\delta x \sim R \;\sqrt{Re^{-1}}$, slightly different from the commonly adopted model for the layer thickness of Rouse and Howe^20^, where $\delta x \sim R \;\sqrt{Re^{-7/8}}$. We demonstrate that the predicted streaming current, $I_{s}$, using this layer thickness, perfectly coincides with the experimental results over four orders of magnitude difference in streaming current, for different nozzle hole diameters, surface treatments and liquid types. We further showed how the charging efficiency changes with salt concentration and pH.

We demonstrated that the charging can have a significant impact on the droplet size and spray plume dynamics. Especially in the case of sprays produced by jets, coalescence can have a considerable impact on the final droplet size, and therefore, the stopping distance of the spray plume. We showed that when droplets are small enough, the self-charging can suppress coalescence and lead to a diverging spray plume. Using computer simulations and a simple model for the electrostatic repulsion, the divergence of the spray cloud can be accurately predicted.

As the streaming current scales linearly with the zeta potential, $\zeta$, the charging efficiency is strongly affected by the surface modification and type of liquid used. Silanol groups of the silicon dioxide surface act as a weak acid in water, releasing protons from the nozzle surface, resulting in a positively charged jet. In accordance with this picture, we showed that the application of a silane coating with positively charged end-groups, leads to a switch in polarity of the streaming current. Interestingly, the use of anhydrous methanol or ethanol gives a positive zeta potential, while when mixed with water gives a negative zeta potential. A complete description of the charging then requires being able to predict the zeta potential given a certain liquid and nozzle surface.

## Data Availability

The datasets used and/or analyzed during the current study are available from the corresponding author upon reasonable request.
